# Clinical implications for the association of psoriasis and multiple sclerosis: an observational study

**DOI:** 10.1007/s10072-024-07616-3

**Published:** 2024-06-01

**Authors:** Giuseppina Miele, Maddalena Sparaco, Elisabetta Maida, Floriana Bile, Luigi Lavorgna, Simona Bonavita, Eleonora Ruocco

**Affiliations:** 1 Department of Advanced Medical and Surgical Sciences, University of Campania Luigi Vanvitelli, Naples, Italy; 2 Dermatology Unit, Department of Mental and Physical Health and Preventive Medicine, University of Campania “Luigi Vanvitelli”, Naples, Italy

**Keywords:** Multiple Sclerosis, Psoriasis, Disease modifying treatment, anti-CD20

## Abstract

**Background:**

Multiple sclerosis (MS) and psoriasis (PsO) are distinct chronic autoimmune conditions with varying impacts on patients' lives. While the co-occurrence of MS and PsO has been reported, the underlying pathogenic link remains unclear. This study aimed to investigate the prevalence of PsO in a MS outpatient clinic population and explore the potential interplay between these conditions.

**Methods:**

316 MS patients who had at least one visit at our MS center in the last year, were selected from our outpatient MS Clinic electronic database and were e-mailed in August 2023 and inquired about a previous diagnosis of PsO. Demographic and MS history data were retrospectively gathered for two groups: MS patients without and with PsO. Information about MS phenotype, Expanded Disability Status Scale (EDSS) score at the diagnosis and at last follow-up, disease modifying therapy (DMT) were collected retrospectively from our MS data set. PsO diagnosis was confirmed by an experienced dermatologist and severity was assessed with the Psoriasis Area and Severity Index (PASI).

**Results:**

Among 253 respondents, 5.85% reported a PsO diagnosis that was confirmed after the dermatological evaluation Among patients with psoriasis 66.67% had progressive course of MS (*p =* 0.032) and the onset of PsO typically occurred after MS diagnosis. 9 out 15 patients had a PASI score of 0 and 6 are currently undergoing treatment with an anti-CD20 therapy. Notably, a subset of our patients were on anti-CD20 therapy and did not experience a worsening of dermatological symptoms.

**Discussion and conclusion:**

The prevalence of PsO in our outpatient MS population aligns with previous studies. Treatment approaches should be tailored to individual patient needs, emphasizing collaboration between neurologists and dermatologists. Medications like dimethyl fumarate, effective in both conditions, could be considered. The data from our study also suggest that anti-CD20 therapy may be a viable option for some patients with concurrent MS and mild PsO, without a significant worsening of dermatological symptoms. Further research is needed to elucidate the complex relationship between MS and PsO and to develop more effective therapeutic strategies for patients with both conditions.

## Introduction

Multiple sclerosis (MS) and Psoriasis (PsO) are two chronic, lifelong conditions that can significantly impact the quality of life, causing various physical and emotional challenges. Although both conditions have a dysimmune origin, their effects on affected individuals differ substantially [1].

MS, an autoimmune disease, primarily affects the central nervous system, involving several functional systems and leading to a range of symptoms, such as difficulties with balance and coordination, motor and sensory disturbances, sphincter problems, visual and cognitive impairments [2].

PsO is an inflammatory skin condition that causes red, scaly patches of the body skin. Psoriatic lesions derive from an acceleration of the epidermal turnover driven by a dysfunction of the immune system. The new cells build up on the surface of the skin, leading to the red, scaly patches typical of PsO [3].

While it is not uncommon to find reports associating MS and PsO in the medical literature, a definitive pathogenic link between the two remains unclear and contradictory [1].

The co-occurrence of MS and PsO could potentially stem from shared genetic [4–6] and environmental factors [7, 8] that may contribute to a dysfunctional immune system response.

In our study we aimed at investigating the prevalence of PsO in our MS outpatient population and to review the literature to shed light on the possible interplay between these two conditions.

## Methods

In August 2023, MS patients of our electronic data base have been selected according to the following criteria: availability of at least one visit in the last year, regular follow-up visits (with a minimum of 1 visit/year), complete data collection during follow-up; each patient fulfilling the above criteria was e-maileda link including a consent form for data processing and participation in the study according to the Declaration of Helsinki and the specific question “Have you ever been diagnosed with PsO by a dermatologist?”. Then MS people were divided into two groups without and with Pso, and for all of them we collected demographic (age and sex), the presence of other autoimmune conditions and MS history data [age at onset, MS phenotype, disease duration, current and previous disease modifying therapy (DMT), wash-out time, Expanded Disability Status Scale (EDSS) score at diagnosis and at the last follow-up visit in the dataset] were collected retrospectively from our electronic database. MS phenotype was defined as RR (relapsing remitting) without or with a progressive course [9].

People with MS who declared to have been diagnosed with PsO were called back for a dermatological outpatient visit in our Institution to confirm the diagnosis, and to collect PsO data (age at the onset, treatment done in the past) and to evaluate the severity of PsO at the moment. The PsO Area and Severity Index (PASI) was used to assess disease severity. When using the PASI, psoriatic plaques are graded based on three criteria: redness, thickness, and scaliness and severity is rated for each index on a 0-4 scale (0 for no involvement up to 4 for severe involvement). The body is divided into four regions comprising the head, upper extremities, trunk, and lower extremities. The highest potential PASI score is 72. A PASI score of ≤10 indicates mild PsO, while scores between 10 and <20 are associated with moderate PsO, a PASI score of ≥20 indicates a severe PsO [10].

Ethical approval for this study was obtained from the ethical committee of the University of Campania “Luigi Vanvitelli” (prot. 0014460).

## Statistical analysis

Continuous variables were presented as mean and standard deviation (SD), and categorical variables were presented as number and percentage. The Shapiro-Wilk test was used to evaluate the distribution of continuous variables for normality. To compare MS subjects with and without PsO, proportions were compared between groups using the Chi-square test or Fisher's exact test, while means were compared using the t-test or the Wilcoxon non-paired test. Finally, univariate and multivariate (adjusted for age and sex) logistic regressions were applied to evaluate the correlation between the diagnosis of PsO and MS demographic and clinical variables. Statistical significance was set at a *p*-value < 0.05. We used Stata/BE 17.0 for statistical analysis.

## Results

Out of 410 MS patients we have selected 316 subjects who had undergone at least one visit at our MS center in the last year . Out of 316 patients 253 answered the link sent via e-mail, resulting in a 80% response rate. 15 out of 253 (5.85%) stated they have been diagnosed with PsO. Other autoimmune conditions observed in our cohort were: autoimmune thyroid disorders in 9.09% (23/253), asthma in 2.76% (7/253), and celiac disease in 0.79% (2/253) cases. Additionally, type 1 diabetes mellitus and inflammatory bowel disease were identified in 0.79% (2/253) and 0.39% (1/253) of cases, respectively.

Comparing MS subjects without and with PsO, we found no significant differences in demographic and clinical characteristics between the two groups. The mean age for those without PsO was 47.86 years (SD = 12.64) and 51.54 years (SD = 12.37) for those with PsO, (*p =* 0.2739). Sex distribution was 68.49% females in the non- PsO group and 53.3% in the PsO group (*p =* 0.224). The mean age at MS onset was 29.58 (SD = 10.13) years for non- PsO patients and 30.38 years (SD = 11.35) for those with PsO (*p =* 0.7695). The duration of the disease was similar between the groups (18.28±10.19 years for non- PsO vs. 21.17±14.23 years for PsO, *p =* 0.3005). The EDSS scores, both at diagnosis (2.43±0.69 for non- PsO vs. 2.8±1.11 for PsO, *p =* 0.1568) and current scores (4.17±1.97 for non- PsO vs. 4.9±1.8 for PsO, *p =* 0.1611), were not statistically different (and, respectively).

Correlation analysis between having PsO and MS course showed that 61.34% patients without PsO, had a RR and 38.66% had progressive MS course. MS patients distribution according MS course, showed that, among patients with PsO, a higher proportion, (66.67%) had progressive MS, and 33.33% a RR course(*p =* 0.032). This significant difference was confirmed at the univariate logistic regression (*p*=0.04), even when adjusting for sex (*p*=0.043) but not adjusting for age (*p*=0,073).

The analysis of DMT distribution in our study population showed that 35.29% patients without PsOand 60.00% of patients with PsO were receiving B cells depleting treatments (*p*=0.053). Concerning the subjects with PsO, the mean PASI score for the patients who attended the dermatological outpatient visit was 1.2 (SD 2.53). 9 out of 15 patients at the time of the dermatological visit had a PASI of 0, meaning they did not have active psoriatic lesions, while 6 out 15 had a PASI < 10 indicating a mild disease. Among 9 patients who had a PASI score of 0, 6 are currently undergoing treatment with an anti-B-cells depleting therapy.

The mean age at PsO onset was about 4 years later than the onset of MS, and approximately three-fourth of patients developed PsO after MS.

Among the 15 patients with both MS and PsO, 5 have experienced a monophasic PsO and 10 out 15 had relapse of PsO. 4 out of 5 these patients with monophasic course were on ocrelizumab treatment (80%), while 1 patient was receiving fingolimod. Only one of these 5 patients had PsO onset before the MS diagnosis.

Data about MS and PsO characteristics are shown in Tables 1 and 2, respectively. In Table 3 are shown the differences between MS patients without and with PsO.

**Table 1:** Demographic and Multiple Sclerosis characteristics

*N patient*	*Sex*	*Age (y)*	Age at onset *(y)*	Disease duration (y)	EDSS at diagnosis	Last EDSS	N of DMTs	Previous DMTs	Ongoing DMT	Treatment duration *(y)*
*n. 1*	*F*	25,38	16,16	9,21	1	2,5	2	INF; Dimetilfumarate	Natalizumab	2,24
*n. 2*	*F*	58,52	14,28	44,24	2,5	6	2	Teriflunomide, Ocrelizumab	Ocrelizumab	2,37
*n. 3*	*F*	64,36	60,06	4,30	6	6	1	Ocrelizumab	Ocrelizumab	3,52
*n. 4*	*M*	53,24	31,51	21,72	2,5	5	2	Teriflunomide, Ocrelizumab	Ocrelizumab	2,90
*n. 5*	*M*	43,75	36,28	7,46	4,5	6	2	Alemtuzumab; Ocrelizumab	Ocrelizumab	1,79
*n. 6*	*F*	36,26	25,04	11,21	4,5	6	2	Fingolimod, Ocrelizumab	Ocrelizumab	3,51
*n.7*	*M*	51,98	38,76	13,22	5	6	2	Fingolimod, Ocrelizumab, Ofatumumab	Ofatumumab	0,77
*n.8*	*M*	60,49	25,26	35,23	2,5	4,5	2	Glatiramer Acetate Ocrelizumab	Ocrelizumab	0,47
*n.9*	*F*	52,91	34,68	18,22	2,5	5	2	Fingolimod Ocrelizumab	Ocrelizumab	0,88
*n.10*	*F*	48,06	30,84	17,22	2,5	3	1	Fingolimod	Fingolimod	8,21
*n.11*	*M*	63,96	32,73	31,23	6	6	1	Ocrelizumab	Ocrelizumab	4,47
*n.12*	*F*	44,68	34,46	10,21	1,5	2	1	Dimetilfumarate	Dimetilfumarate	6,87
*n.13*	*F*	70,46	16,38	54,08	1,5	6	1	Glatiramer Acetate	Glatiramer Acetate	6,70
*n.14*	*M*	36,19	22,97	13,22	2	3	1	Fingolimod	Fingolimod	9,21
*n.15*	*M*	59,24	36,0	23,22	4,5	7,5	2	Fingolimod; Ocrelizumab	no DMT	x

*DMT* disease modifying therapy, *EDSS* Expanded Disability Status Scale, *MS* multiple sclerosis

**Table 2:** PsO characteristics

*N patient*	*Sex*	*Age (y)*	Age at Onset	Description of lesion	Locations of lesions	Treatment	PASI score	Disease course
*n. 1*	*F*	25,38	22,38	small non-itchy erythematous-desquamative patches	Scalp	topical treatment which resulted in a clinical improvement of the dermatosis	0.4	2021: recrudescence in the same location improved after topical treatment
*n. 2*	*F*	58,52	28,52	small non-itchy erythematous-desquamative patches	elbows	topical preparations based on corticosteroids, which resulted in a clinical improvement of the dermatosis	0	Sporadic flare-ups over the next 10 years with an optimal response to topical steroid treatment.
*n. 3*	*F*	64,36	60,36	small non-itchy erythematous-desquamative patches	elbows	topical preparations based on corticosteroids, which resulted in a clinical	2,4	About 2-3 flare-ups per years with an optimal response to topical steroid treatment.
*n. 4*	*M*	53,24	49,24	small non-itchy erythematous-desquamative patches	elbows	topical preparations based on corticosteroids, which resulted in a clinical improvement of the dermatosis	0	No recurrence
*n. 5*	*M*	43,75	36,75	small erythematous-desquamative patches	elbows and knees	topical preparations based on corticosteroids and vitamin D derivatives, which resulted in a clinical improvement of the dermatosis	9.6	December 2021: recrudescence always in the same locations and also extending to the sternal region accompanied by intense itching (treated with topical preparations based on corticosteroids and vitamin D derivatives, resulted in a clinical improvement).
*n. 6*	*F*	36,26	6,26	Small erythematous-desquamative patches	elbows and knees	topical preparations based on corticosteroids, which resulted in a clinical improvement of the dermatosis	0	No recurrence
*n.7*	*M*	51,98	18,98	small non-itchy erythematous-desquamative patches	elbows	topical steroid therapy which resulted in a clinical improvement	2,4	Until the diagnosis of multiple sclerosis (2014), PsO alternated periods of clinical remission, with periods of recrudescence always in the same locations. After 2014, PsO remained stable, not resulting in a clinical worsening.
*n.8*	*M*	60,49	40,49	small erythematous-desquamative patches	intergluteal cleft	topical steroid therapy which resulted in a clinical improvement	0	Sporadic flare-ups over the following 10 years with an optimal response to topical steroid treatment.
*n.9*	*F*	52,91	46,91	small erythematous-desquamative patches	elbows	topical steroid therapy which resulted in a clinical improvement	0	No recurrence
*n.10*	*F*	48,06	30,06	small erythematous-desquamative patches	Scalp and elbows	topical preparations based on corticosteroids and vitamin D derivatives, oral supplements, which resulted in just a slight clinical improvement of the dermatosis	2.8	Since 2005, the dermatosis has never completely disappeared. Currently under topical treatment.
*n.11*	*M*	63,96	33.96	small non-itchy erythematous-desquamative patches	Scalp	topical preparations based on corticosteroids and vitamin D derivatives which resulted in a clinical improvement	0	No recurrence
*n.12*	*F*	44,68	36.68	small erythematous-desquamative patches	Heels and tibial regions	topical steroid therapy which resulted in a clinical improvement	0	Sporadic flare-ups over the following 3 years with an complete response to topical steroid treatment.
*n.13*	*F*	70,46	66.46	small erythematous-desquamative patches	elbows	topical preparations based on corticosteroids and vitamin D derivatives which resulted in a clinical improvement	0	No recurrence
*n.14*	*M*	36,19	26.19	small non-itchy erythematous-desquamative patches	elbows and tibial regions	topical steroid therapy with disappearance of the clinical manifestations	0	In 2016: exacerbation of PsO in the elbows, tibial regions, and back region, reduced with topical treatment of steroid and vitamin D derivatives.
*n.15*	*M*	59,24	29.24	small non-itchy erythematous-desquamative patches	Scalp	topical steroid therapy with disappearance of the clinical manifestations	0.4	Sporadic flare-ups over the following 25 years with complete response to topical steroid treatment.

*PsO* psoriasis; *PASI* (Psoriasis Area Severity Index)

**Table 3:** Differences between MS patients without and with PsO

Variable	Without PsO (238)	With PsO (15)	P Value
Age, years	47.86 ± 12.64	51.54 ± 12.37	0.2739
Female sex, n (%)	163 (68.49)	8 (53.30)	0.224
Age at Onset, years	29.58 ± 10.13	30.38 ± 11.35	0.7695
EDSS at Diagnosis, years	2.43±0.69	2.8±1.11	0.1568
Current EDSS	4.17±1.97	4.9±1.8	0.1611
Disease Duration	18.28±10.19	21.17±14.23	0.3005
RR course, n (%)	140 (61.34)	5 (33.33)	0.032*
Current use of B cell depleting treatments, n (%)	84 (35.29)	9 (60.00)	0.054

*EDSS* Expanded Disability Status Scale, *MS* multiple sclerosis, *RR* relapsing remitting ;*p<0,05.

### Discussion

PsO is a chronic autoimmune disorder with a prevalence of 3% in the general population, while an estimated 2.8 million people worldwide live with MS [11].

While several studies have shown that the frequency of PsO in patients with MS does not differ from that in the general population [12, 13] , a systematic review conducted in 2015, aimed at determining the occurrence and prevalence of comorbid autoimmune diseases in MS, found the prevalence of PsO in MS patients ranging from 0.39% to 7.7% [14] (ref). This aligns with the 5.85% prevalence of PsO observed in our study population.

Moreover, several investigations have been undertaken to determine if there is an association and potential risk of developing one condition when already affected by the other [15, 16]. However, to date, the results are conflicting, leaving the exact nature of this relationship unclear. Notably, an initial observation in 1989 reported a higher prevalence of PsO among Polish MS patients compared to controls [17], a finding that has been supported by subsequent and more recent studies in various MS populations [2, 4]. Conversely, other research involving larger samples of MS patients has failed to establish a convincing link between MS and PsO, highlighting the necessity for further research in this area [12, 14].

Nevertheless, although the association between the two conditions has not been fully elucidated, both diseases share an autoimmune pathogenesis, and certain mechanisms appear to be correlated. In both MS and PsO, T cells play a crucial role in the immune response. Although recent evidence suggests a significant role for B cells in the MS pathogenesis, T cells are widely considered to be the major contributors to inflammatory demyelination [18]. Moreover, recent extensive literature has suggested a close association between T cells and the pathogenesis of PsO [19]. In addition to being both T-cell mediated conditions, MS and PsO also share similar cytokine pathways [20, 21].

The IL-23/IL-17 axis is important in the development of both MS and PsO. IL-23 contributes to the expansion of Th17 cells, which are pro-inflammatory cells in both diseases. In PsO, the inflammatory response is driven by Th17 cells, while in MS, Th17 cells infiltrate the central nervous system and produce IL-17 [21]. Moreover, TNF-alpha is another key cytokine involved in both diseases. Increased levels of TNF-alpha are found in the affected areas of both MS and PsO, emphasizing the role of these inflammatory mediators [22].

Beyond the mere co-occurrence of the two autoimmune diseases, we explored whether certain demographic and clinical features could be associated with the presence of PsO in people with MS. We did not observe a significant difference in demographics and clinical characteristics, except for a higher frequency of progressive forms of MS in the group with PsO, though this difference was not confirmed after adjusting the analysis for age suggesting age as a potential confounding factor.

The treatment of a patient with both MS and PsO depends on the specific needs and symptoms of the patient. Since both conditions are complex and involve the immune system, sharing some autoimmune processes, a tight collaboration between the MS neurologist and the dermatologist is fundamental to plan a personalized treatment, possibly targeting both diseases. Regarding MS, several DMTs may be prescribed to reduce the occurrence of relapses and disease progression. DMTs include injectable, oral, and infusion therapies (i.e., monoclonal antibodies) with different mechanisms of action [23]. Among PsO-specific therapies, topical medications such as corticosteroid creams or ointments, or calcipotriol, may be prescribed. While, in more severe cases, systemic medications such as methotrexate, acitretin, or biologic drugs like TNF-alpha inhibitors or IL-17 or IL-23 inhibitors may be necessary [24]. Fumarate, specifically dimethyl fumarate, is a medication that has been used in the treatment of both MS and PsO [25]. In the context of MS, dimethyl fumarate has been approved for relapsing forms of MS. It is thought to have immunomodulatory effects by reducing inflammation and oxidative stress in the central nervous system by activating the pathway of the nuclear factor erythroid 2 -related factor 2, which leads to the increased production of antioxidants and anti-inflammatory molecules [26]. In the case of PsO, oral dimethyl fumarate, as a first-line systemic therapy, has shown efficacy in reducing psoriatic skin lesions and improving symptoms. The exact mechanism of action in PsO is not fully understood, but it is believed to involve modulation of the immune system responses and inhibition of pro-inflammatory cytokines, as in MS [27].

Based on our findings, patients with both conditions have undergone various DMTs for MS over the years; patients who had more frequent PsO exacerbations (10 out of 15) received different DMTs with different therapeutic switches. Apparently in our MS population, there was no evident temporal relationship between therapeutic changes for MS and PsO exacerbation, except for patient number 5, who manifested the first signs of PsO two months after the first infusion of alemtuzumab. This data strongly contrasts with what has been described in the literature, as even though the underlying biology of the response in PsO is not well understood, it is primarily mediated by T Cells which alemtuzumab effectively depletes [28].

Pragmatic treatment approaches for patients with MS and coexisting PsO are already reported in literature. More in details, general contraindications for people with MS is the treatmeant with TNFα blockers that are effectively utilized in PsO, yet have been shown to exacerbate or induce demyelination [22 ]. On the other hand people with MS with mild/moderate disease activity and coexisting PsO could be prescribed with Dimethyl Fumarate, while in case of high disease activity, S1PR modulators (fingolimod, siponimod, ozanimod) or alemtuzumab could be considered.

The recent approach on DMTs selection for people with MS points toward the use of high efficacy therapy since the beginning of the disease [28]. Conflicting data exist regarding the use of B cells depleting agents (anti-CD20 monoclonal antibodies) in patients with both MS and PsO, as the use of anti CD20 is discouraged due to some case reports though not generalizable to the whole patients population. A temporal correlation has been noted between PsO onset and rituximab use in patients with rheumatoid arthritis, showing psoriatic lesions across diverse areas (scalp, knees/thighs, elbows/arms/trunk/onycholysis, scalp/extensor surfaces, arms/thighs, trunk/arms) [29]. Two case reports showed a link between ocrelizumab treatment for MS and PsO onset. The first case report in 2018 detailed mild PsO on the trunk of a 68-year-old MS patients within six months of ocrelizumab initiation and no recommendation was made to discontinue the next infusion of ocrelizumab [15]. , The second case in 2023 described a 34 -year-old patient, who experienced two months after the initial full dose of Ocrelizumab severe itchy, patchy lesions, diagnosed as guttate PsO. Despite topical steroids and UV therapy, PsO worsened, evolving into psoriatic arthritis. Shifting to IL-17 antagonist (secukinumab) yielded positive results and ocrelizumab was stopped [30]. These two cases suggest that in comorbid MS and PsO deserve meticulous evaluation of the severity of both condition to prioritize treatment for the better patient welbeing. Mild PsO in the first case justified continuing ocrelizumab, yielding subsequent clinical benefits for MS. Conversely, severe PsO in the second case prompted ocrelizumab discontinuation, opting for PsO therapy optimization despite MS stability. Additionally, it is noteworthy that, although in our cohort of patients treated with ocrelizumab there was no worsening of PsO, several literature reports associate anti-CD20 antibodies therapies with the onset of PsO. Despite the risk management plans of anti-CD20 treatments in MS does not include skin-related adverse reactions such as PsO, the literature describes PsO rash occurrence following ocrelizumab administration. Specifically, these cases are highlighted in the recent 2024 literature review, where 2 patients out of 8 reported cases of new-onset skin events, developed PsO dermatitis after initiating ocrelizumab. The PsO rash appeared in a interval between the second infusion aof OCR and 2.5 years after treatment start. The same authors assert that establishing a causal relationship is challenging; indeed, it is difficult to discriminate between a a drug reaction, the unmasking of a previous vulnerability, or a secondary autoimmunity [31]. Some studies have also highlighted that the DMTs most strongly associated with the onset of new PsO rashes in patients with a sole diagnosis of MS are anti-CD20 agents, particularly rituximab more than ocrelizumab [32]. Therefore, although we cannot exclude skin-related adverse reactions due to ocrelizumab, we can at least establish that in our patient cohort presenting both MS and a preexisting PsO condition, no worsening or exacerbation of PsO was observed using an anti-CD20 treatment.

Although our data do not allow generalization and do not provide sufficient information on how to proceed with MS DMTs choice in this peculiar population, 9 out of 15 MS patients with coexisting PsO are currently being treated with an anti-CD20 (8 ocrelizumab and 1 ofatumumab) and none had a significant worsening of PsO, except for some sporadic PsO recrudescence improved with topical treatment, in the face of improvement or stabilization of MS-related symptoms. The PASI index, used for assessing the severity of PsO, resulted in 0 among 9 out 15 MS patients with PsO and among them 6 were receiving anti-CD20 therapy and showed no evidence of PsO exacerbation from anti CD20 start.

These findings suggest there is no evidence of a detrimental effect of anti-CD20 agents on PsO progression. Therefore, opting for an anti-CD20 agent in patients with both MS and PsO could be justified when there is a need to manage more aggressive or active MS. This reflects a strategic decision to prioritize treatment based on the relative urgency of the MS condition over PsO.

The study's limitations encompass a small sample size, possible response bias due to a 20% non-response rate, reliance on self-reported data susceptible to recall bias, a cross-sectional design hindering causal conclusions, scant demographic details excluding factors like race or socioeconomic status while offering valuable insights, these constraints underscore the necessity for more extensive, diverse investigations to enhance our understanding of this complex relationship.

Although the study’s limitations, we believe that this dataset provides insights for neurologists to make informed decisions regarding the optimal treatment for MS. In case of clinically and radiologically highly active MS, it encourages clinicians to prioritize the most suitable treatment for MS control, also anti CD-20, for each patient, irrespective of the concurrent presence of PsO, that does not appear to be worsened by these treatments.

Currently, there is no literature suggesting the superiority of one treatment over another in treating both diseases therefore the choice of a DMT for people with MS and comorbid PsO should be based on each patient's MS characteristics and severity of the disease.

Nevertheless, we strongly advocate for collaboration between neurologists and dermatologists to ensure comprehensive monitoring and care for both conditions. Further studies are needed to gain a better understanding of the relationship between these two autoimmune disorders and to possibly develop unique effective treatment targeting both diseases.
